# Synergistic Effects of Arsenic Trioxide and Radiation: Triggering the Intrinsic Pathway of Apoptosis

**DOI:** 10.18869/acadpub.ibj.21.5.330

**Published:** 2017-09

**Authors:** Kave Moloudi, Ali Neshasteriz, Arshad Hosseini, Nazila Eyvazzadeh, Mehdi Shomali, Samira Eynali, Elahe Mirzaei, Asaad Azarnezhad

**Affiliations:** 1 Radiation Sciences Department, Faculty of allied Medicine school, Iran University of Medical Sciences, Tehran, Iran; 2 Radiation biology research center, Iran University of medical sciences, Tehran, Iran; 3 Department of Medical Biotechnology, Faculty of allied Medicine, Iran University of Medical Sciences, Tehran, Iran; 4 Radiation Research Center, Faculty of Paramedicine, AJA University of Medical sciences, Tehran, Iran; 5 Radiology Department, Faculty of allied Medicine, Tehran University of Medical Sciences, Tehran, Iran; 6 Medical physics and Biomedical Engineering Department, school of Medicine, Tran University of Medical Sciences, Tehran, Iran; 7 Microbiology Department, Faculty of Science, Islamic Azad University, Tehran, Iran; 8 Cellular & Molecular Research Center, Kurdistan University of Medical Sciences, Sanandaj, Iran; 9 Department of Medical Genetics, School of Medicine, Tehran University of Medical Sciences, Tehran, Iran

**Keywords:** Arsenic trioxide, Radiation, Apoptosis, Glioblastoma, Spheroids

## Abstract

**Background:**

Arsenic trioxide (ATO) has been reported as an effective anti-cancer and a US Food and Drug Administration (FDA) approved drug for treatment of some cancers. The aim of this study was to determine the underlying apoptosis molecular and cellular mechanisms of ATO in the presence or absence of ionizing radiation (IR) *in vitro* in the glioblastoma multiforme (GBM) cell line, U87MG.

**Methods:**

Cells were treated by different concentrations of ATO either in presence or absence of IR. Viability and apoptosis pathway of both treated and control groups were evaluated using MTT assay and the expression analysis of *Bax*, *Bcl-2*, and *caspase-3* genes, respectively. All treatments were performed on 100-μm diameter spheroids.

**Results:**

Results showed a significant reduction in the survival of the cells in all treated groups. As expected, cell survival was much less in combination treatment than treatment with only ATO. Moreover, combination therapy made *Bax* and *caspase-3* up-regulated and *Bcl-2* down-regulated.

**Conclusion:**

ATO and radiation had a synergistic apoptotic effect on GBM cells by up-regulation of *caspase-3* and alteration of the *Bax*-*Bcl-2* balance; therefore, ATO may act as a potential anti-cancer agent against GBM cells through triggering the mitochondrial pathway of apoptosis.

## INTRODUCTION

In spite of many advances, cancer is one of the major problems in the area of public health[1]. Gliomas are the most common type of brain tumors and responsible for 15% of brain tumors[2]. Unfortunately, these malignant tumors are poorly diagnosed and treated. The mortality rate of glioblastoma multiforme (GBM) is about 12,000 per year in the USA, and three per 100,000 people develop the disease a year[3,4]. The disease is more frequent in males and generally occurs in the sixth decade of life[5]. The standard treatment regimen of GBM includes surgery, followed by radiotherapy[5]. Nonetheless, due to innate aggression, chemo/radiation resistance, hypoxia, and blood-brain barrier, glioblastoma remains difficult to treat, and survival is only 14 months after diagnosis[6,7]. Unfortunately, the brain is very damageable against conventional therapy, and the total ionizing radiation (IR) dose essential to control the tumors is much more than normal brain tissue tolerance[8]. Radiotherapy is the method of choice to treat about 50% of cancers. However, its application and efficiency are limited because of the mentioned reasons. Thus, complementary methods and agents can be utilized to enhance sensitivity to radiotherapy and avoid such unfavorable outcomes.

With the hope of curing cancer either by eliminating a tumor or preventing cancer recurrence, X-ray therapy has been used in combination with curative intent[9]. Literature has revealed that the combination of IR with ATO (arsenic trioxide) is a more effective treatment[10]. ATO is a Food and Drug Administration approved drug employed for centuries to treat a variety of conditions, including psoriasis, syphilis, and rheumatism, and its derivatives have been used as anti-cancer agents for many years[11,12]. It has recently been investigated that ATO has an effective role in treatment of acute promyelocytic leukemia (APL)[13]. Chiu *et al*.[14] have shown that ATO enhances the radiation sensitivity of androgen-dependent and -independent human prostate cancer cells via increasing reactive oxygen species level and autophagy. Some *in vitro* studies have disclosed that ATO exerts its anti-cancer effects through inhibition of tumor growth by increasing the levels of reactive oxygen species and inducing apoptosis[14,15]. Also, apoptotic and non-apoptotic cell death is induced by radiation therapy. DNA damage response system is the biological consequences of IR, leading to the activation of apoptosis[16]

Apoptosis is a programmed cell death process in multicellular organisms and its deficiency is implicated in a wide range of diseases. One mechanism of anti-cancer function of IR and chemotherapy drugs is the induction of apoptosis[17]. Down-regulation of *Bcl-2* and up-regulation of *caspase-3* have been suggested as the mechanisms of ATO for inducing apoptosis[18,19]. Furthermore, in an ATO-dependent apoptotic process, the mitochondrial permeability is directly affected and the reactive oxygen species would be generated[20]. There is some information on the ability of ATO in inducing extrinsic pathway mediated by Fas cell surface death receptor (Fas) and caspase-8 activation in acute megakaryocytic leukemia[21]. Although the possible effects of ATO have been reported in some cancers such as APL, the exact mechanism of ATO function in GBM is not fully determined.

We hypothesized that ATO alone or in combination with IR would potentiate the efficacy of each other by increasing the apoptosis rate of cancerous cells. Therefore, an experiment was designed based on a three-dimensional culture system to assess the apoptotic effects of ATO alone and in combination with IR on spheroids of U87MG cell line in cellular and molecular level *in vitro*.

## MATERIALS AND METHODS

### Cell culture

Human glioblastoma cell line, U87MG, maintained in MEM (GIBCO, USA) supplemented with 10% FBS (GIBCO, USA), 500 U/ml penicillin (Sigma, USA), and 200 mg/L streptomycin (Biowest, USA) was obtained from Pasteur Institute of Iran (Tehran, Iran). Cells were cultured as a monolayer at a density of 200,000 cells/ml in T-25 tissue culture flasks (SPL, USA) in an incubator (Memmert, Germany) in 5% CO_2_ humidified atmosphere at 37°C. To achieve 70-80% of confluency and ensure to maintain cells in the logarithmic phase of growth, cells were trypsinated (1 mM EDTA/0.25% Trypsin (w/v), Sigma, USA) and then sub-cultured. Cell viability >97% was confirmed by trypan blue staining. Spheroids of uniform size and diameter from 450 to 550 µm were produced using the liquid overlay technique. U87MG cells (5×10^5^) were seeded in 100-mm dishes coated with a thin layer of 1% agar (Bacto Agar, Difco, Detroit, MI, USA) in 10 ml MEM supplemented with 10% FBS and incubated in the above described condition for one month. Half of the culture medium was replaced with fresh medium twice per week.

### Drug-radiation treatment

ATO (MW: 164.84 g/mol) (KANTO CHEMICAL Co, Japan) was dissolved in 1M NaOH, as the stock solution, and diluted to 10 mM with double-distilled water prior to use. Treatment of U87MG spheroids was performed with ATO either with or without X-ray. In combination, U87MG spheroids in approximately 100 μm of diameters were treated by ATO (2 μM and 5 μM diluted in growth medium, pH 7.0–7.4) for one volume doubling time (VDT, 54.7 h). IR was then performed with 6 MV X-rays using a linear accelerator (Linac 600, GMV; Varian Medical Systems; USA) at a dose rate of 4 Gy/min. An additional 2 cm tissue-equivalent bolus was placed on the top of a culture flask to ensure electronic equilibrium, and 10 cm of tissue-equivalent material was placed under the flask to obtain full backscatter.

### MTT assay

MTT assay was used to measure the cell viability for ATO treatment alone or in combination with IR. In order to prepare single cells, spheroids were trypsinized with 300 μl trypsin in 5 minutes after one VDT. Then the medium was discarded, and the cells were washed with PBS and 10,000 cell/ml seeded in 96-well polystyrene tissue culture plates. The cells were then placed in a humidified 5% CO_2_ incubator at 37°C for 24 h. After incubation, medium was removed, and 20 μL aliquots of MTT solution (5 mg/ml in PBS, Sigma, USA) were added to each well and re-incubated at 37°C for 4 h. Next, 200 μL of the supernatant culture medium was carefully aspirated, and 200 μL aliquots of DMSO were added to each well to dissolve the formazan crystals, followed by 10-min incubation to dissolve air bubbles. The culture plate was placed on a microplate reader (Biotex, Houston, Texas, USA) and shaked for 20 min, and absorbance was then measured at 570 nm. The amount of color produced is directly proportional to the number of the viable cells. All assays were performed in eight replicates for each concentration, and each assay was repeated at least two times. Cell viability rate was calculated as the percentage of MTT absorption as shown in the following equation: % survival=(mean experimental absorbance)/(mean control absorbance)×100.

### Analysis of cell morphology and apoptosis

Annexin-V-FLUOS staining Kit (Roche Life Science, Germany) and Type Direct fluorescence staining were used for flow cytometric or microscopic analysis of apoptotic cells with membrane alterations (phosphatidylserine translocation) and differentiation of apoptotic from necrotic cells. In this light, different treated groups of spheroid cells were singled and washed with PBS and centrifuged at 180 ×g at 4°C for 5 min. The cells were then incubated in a binding buffer containing Annexin-V-FLUOS and propidium iodide or Annexin-V-Alexa 568 (red dye) at 37°C for 10 min. The stained cells were analyzed by fluorescence microscopy and flow cytometry. Necrotic cells take up propidium iodide and stained orange/green, while apoptotic cells stained green only.

### Quantitative reverse transcription PCR (RT-qPCR)

A commercial RNA isolation kit (Jenabioscience, Germany) was used to extract total RNA from 4×10^6^ cells of all groups (control, 2 µM, 5 µM, 4 Gy, 2 µM+4 Gy, and 5 µM+4 Gy) according to the manufacturer’s instructions. The quality and quantity of extracted RNA samples were assessed using the Nanodrop ND-1000 (Nanodrop technologies, USA). Based on the quality and quantity of extracted RNA, 500 ng-1 μg RNA was converted into cDNA using AccuPower CycleScript RT PreMix (Bioneer, Korea) according to the manufacturer’s instructions. Glyceraldehyde 3-phosphate dehydrogenase (*GAPDH*), *Bax*, *Bcl-2*, and *caspase-3* primer sequences were designed using the Primer3 software (version 0.4.0). *GAPDH* was used as an internal control to normalize the expression of target genes. Primers sequences designed for amplification of the targets are listed in Table 1.

**Table 1:** Nucleotide sequences of the primers used for real-time RT-PCR

Gene	Accession number	Forward primer (5’-3’)	Reverse primer (5’-3’)	Product size (bp)
*Bax*	NM_001291428	GGACGAACTGGACAGTAACATGG	GCAAAGTAGAAAAGGGCGACAAC	150
*Bcl-2*	NM_000633	ATCGCCCTGTGGATGACTGAG	CAGCCAGGAGAAATCAAACAGAGG	129
*Casapse-3*	NM_032991	ATGGAAGCGAATCAATGGACTC	CTGTACCAGACCGAGATGTCA	138
*GAPDH*	NM-001289745	GAGTCAACGGATTTGGTCGT	GACAAGCTTCCCGTTCTCAG	185

### Data analysis

The cycle threshold (CT) values provided by RT-qPCR were used to calculate the relative fold expression according to the 2^-ΔΔCT^ method[22] and REST 2009 software (version 2.0). All experiments were performed in triplicate. Comparison between different groups was performed by using one-way ANOVA and the Dunnett’s multiple test. A value of *P*≤0.05 was considered to be statistically significant. Data were expressed as the mean±SD.

## RESULTS

### Growth curve

**Fig. 1:**
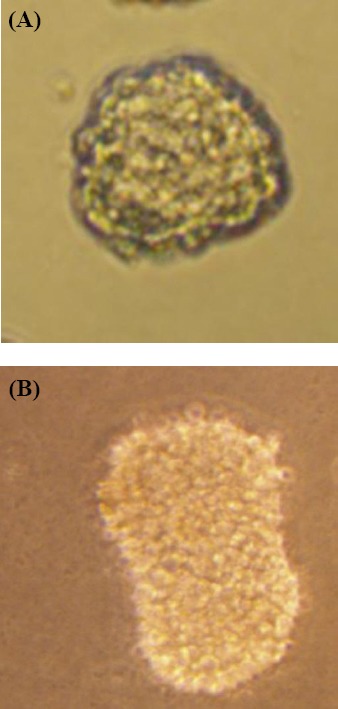
The microscope micrographs of U87MG spheroids. **A)** 100-µm spheroid at day 9; **B)** 300-µm spheroid at day 20.

**Fig. 2:**
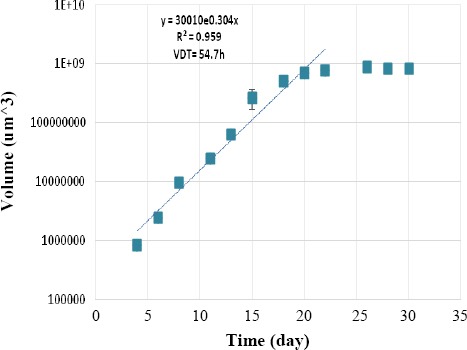
The growth curve of the U87MG cell line in the spheroid cultures. The days 4 to 20 show the log phase of the curve and used to measure the volume doubling time (54.7 h).

Figure 1 shows the microscopic micrographs (Olympus Corporation of America) of spheroids in two different diameters. The growth curve of U87MG spheroids is shown in Figure 2. The VDT calculated from the curve was approximately 54.7 hours, which was consequently applied as the drug treatment time. Treatment with ATO at the presence and/or absence of 4 Gy x-ray was performed on spheroids with 100 μm of diameter after one VDT.

### Viability assessment

**Fig. 3:**
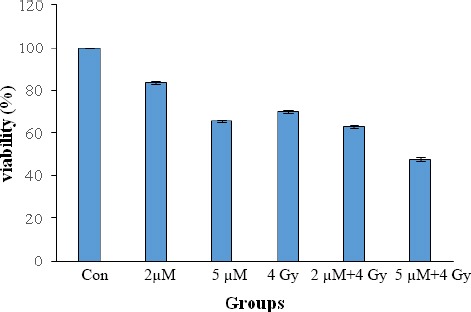
Cell viability of spheroids U87MG cells after treatment with ATO alone or co-treatment with IR for 54.7 h. Cell viability was determined based on the MTT assay results. Each point represents a mean value and standard deviation of three experiments with eight replicates per dose. Cell viability in treated groups were significantly different (*P*≤0.05) compared to the control.

Figure 3 shows the viability rate (%) of the treated groups after one VDT. As shown in the Figure, ATO significantly reduced the viability of U87MG cells. The viability of different groups containing control and the groups treated with 2 µM ATO, 5 µM ATO, 4 Gy, 2 µM+4 Gy, 5 µM+4 Gy was 100%, 83%, 56%, 65.65%, 70.08%, 62.76%, and 47.74%, respectively.

### Real-time PCR results

**Fig. 4:**
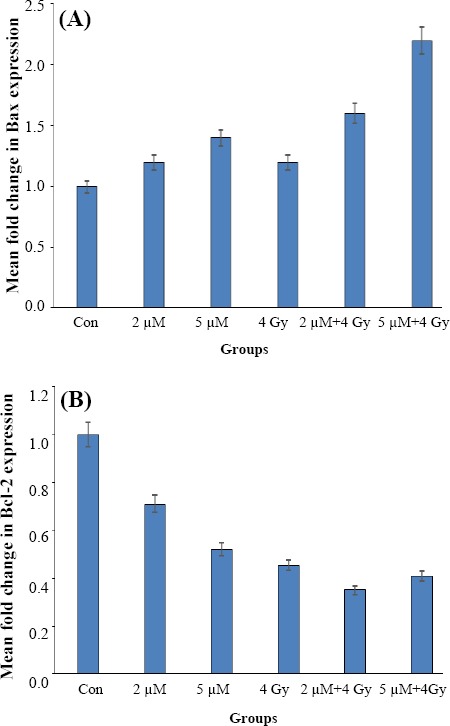
Expression status of *Bax* and *Bcl-2*. (A) Mean fold-change in gene expression level of *Bax* (A) and *Bcl*-2 (B) in U87MG spheroid following treatment by ATO±IR for one doubling time (54.7 h). Each bar represents the mean±SD of the results of three independent experiments.

**Fig. 5:**
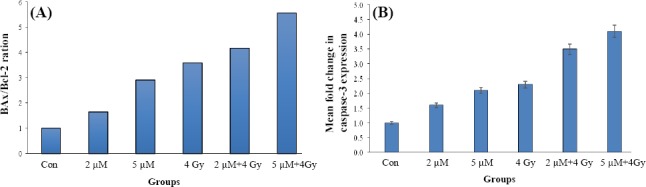
Expression ratio of *Bax/Bcl-2* and *caspase3*. Relative fold expression ratio of *Bax/Bcl-2* (A) and mean fold-change in gene expression level of *caspase-3* (B) and in U87MG spheroids following treatment by ATO±IR for one doubling time (54.7 h). Each bar represents the mean±SD of the results of three independent experiments.

Comparison of the relative mean fold changes in gene expression levels of *Bax*, *Bcl-2*, *caspase-3*, and *Bax*/*Bcl-2* ratio in U87MG spheroids treated by ATO±IR in one VDT (54.7 h) is shown in Figures 4 and 5. Data indicated that the relative expression of *Bax* (Fig. 4A) was increased in all treated samples (*P*≤0.05), as compared to the control group. In combination group (ATO and IR), *Bcl-2* was found to be up-regulated more significantly than single treatment group, indicating the synergistic effect of IR and ATO. According to Figure 4B, the expression of the anti-apoptotic *Bcl-2* gene was decreased in comparison to control group (*P*<0.05). The *Bax*/*Bcl-2* balance shows the degree of vulnerability of the cells to apoptosis. As demonstrated in Figure 5A, the combination of ATO and radiation induced more apoptosis than treatment with ATO or radiation alone. Also, the expression level of *caspase-3* was increased in the presence of ATO and 4 Gy radiation (Fig. 5B). Alternatively, the combination of ATO+IR noticeably far increased the expression of *caspase-3* in corresponding doses of ATO or 4 Gy alone.

### Cell morphology

**Fig. 6:**
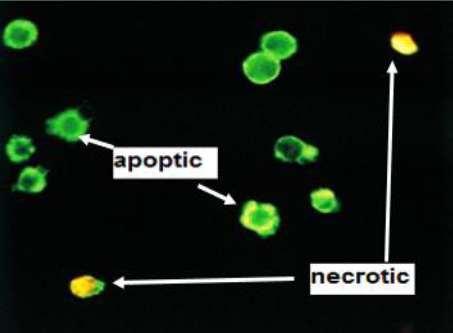
Cell morphology and apoptosis assay. The apoptotic cells are visible in green and can be differentiated from necrotic cells that stain orange/green.

Fluorescence micrographs (Olympus Corporation, USA) and annexin-V-FLUOS staining kit were used to assay cell morphology. As it is shown in Figure 6, the apoptotic cells were visible in green and could be differentiated from necrotic cells after propidium staining.

## DISCUSSION

GBM is the most common and the most malignant form of the glial tumors. Surgery, radiotherapy, chemotherapy, and immunotherapy are the common treatments for cancer in general and GBM in particular[23]. However, these approaches can extend the patients’ survival only for few months[24,25]. Although radiotherapy is one of the widely-used treatments, it faces several disadvantages. The development of adjuvant agents can increase therapeutic effects of radiotherapy and decrease its drawbacks such as normal tissue damage and toxicity[26]. Therefore, a combination of different antitumor treatment modalities is advantageous in limiting the nonspecific toxicity[27]. Elucidation of the cellular and molecular mechanisms of the adjuvants functions is a major step to use them appropriately in clinic. Previous studies have indicated that ATO induces apoptosis and cell death[28,29]; however, its molecular mechanism in glioblastoma remains to be fully understood. These observations prompted us to evaluate the potential anticancer effects of ATO alone or in combination with IR on U87MG cell line and the possible activated molecular pathways of apoptosis.

Our cellular and molecular data revealed that ATO alone and more strongly in combination with IR decreased the cell survival through inducing apoptosis. In consistent with our findings, some phase II clinical trials have also shown that ATO alone has a limited activity and suggested that ATO should be used in combination with other therapeutic modalities such as radiotherapy[30]. Activation of a sensor’ caspase, such as caspase-8 or -9 following with the activation of other ‘effector’ caspases (e.g. caspase-3) is the probable mechanism of the synergism effect of ATO and X-ray that lead to cleavage of a large set of cellular proteins and ultimately destruction of the cell[31].

Recent studies have indicated that low doses (0.5-2 µM) of ATO induce apoptosis and cell cycle arrest in G2/M phases[28,29], whereas high doses (more than IC50) have shown more cytotoxicity and low therapeutic effect. ATO in doses of 1-2 µM has revealed a great effect on APL with a low toxicity and good tolerance[32]. Nevertheless, IC50 of this drug has reported to be 5 µM in several cell lines, such as APL and renal carcinoma[33]. In line with these findings, our results disclosed that ATO induces cell apoptosis and consequently decreases cell growth *in vitro*. MTT assay revealed that ATO decreases cell viability in a dose-dependent manner so that cell survival in 2 µM and 5 µM was 83.56% and 65.65%, respectively. However, ATO combined with IR was more effective, and only 47.74% of the cells survived in exposure to 5 µM ATO+4 Gy IR. Morphologically, apoptosis is firstly characterized by a change in the refractive index of the cell[34], followed by cytoplasmic shrinkage and nuclear condensation. In the present work, cell morphology study showed that ATO alone or in combination with IR possibly decreases cell growth and proliferation via inducing apoptosis.

Apoptosis is the major type of programmed cell death that is crucial for the balance between cell death and cell survival of normal cells; therefore, disturbing this equilibrium can lead to different diseases including cancer[35]. There are two core pathways to induce apoptosis: extrinsic pathway that is triggered by Fas/Fas ligand composite and intrinsic pathway that is mediated by mitochondria[36]. However, the activation of *caspase-3* in final step is common between two pathways[37].

The cell viability was assessed using MTT assay. As it is shown in Figure 3, the combination of 5 µM ATO with 4 Gy radiation had the greatest synergistic effect. It is expected that 5 µM of ATO arrests more cells in G2/M, the cell sensitive phase to radiation; therefore, the cell survival rate was 47.74%. We also found that the survival rate of cells in treatment condition with 2 µM ATO alone and in combination with IR was 83.56% and 62.76%, respectively. As mentioned, radiation and ATO induce apoptosis; hence, it can be expected that the administration of high doses of these agents induces more cell death than low doses. Our data in Figure 3 confirmed this claim.

In this study, we focused on the *Bax*, *Bcl-2*, and *caspase-3* expression that are important biomarkers of the intrinsic pathway of apoptosis. Similar to other investigation[18,38-40], our data showed that ATO has the property to induce the up-regulation of *Bax* and *caspase-3* (1.4 and 2.1folds, respectively) and down-regulation of *Bcl-2* (near to 0.5fold). Therefore, it can be concluded that ATO induces molecular pathway of apoptosis, which confirms our cellular results. *Bax* affects the permeability of mitochondrial membrane, resulting in cytochrome c leakage. This feature helps recruiting and activation of initiator caspase-9, which induces intrinsic pathway of apoptosis through the activation of *caspase-3*. In contrast to *Bax*, *Bcl-2* is a pro-survival factor that provides a balance between cell death and survival. As it has been shown in Figure 5A, our finding confirmed that ATO alone (1.64-2.92fold) and synergistically in combination with IR (4.16-5.56-fold) disturbs this balance in favor to cell death. *Caspase-3* is the other key player that acts at the terminal of apoptosis. According to the expression signature of *Bax*/*Bcl-2*, ATO may activate intrinsic pathway of apoptosis, though *caspase-3* is the milestone of both intrinsic and extrinsic pathways. Dizaji *et al*.[41] have reported that ATO and Silibinin have synergistic effects on human glioblastoma U87MG cell line. The 2-µM ATO decreased the expression of *Bcl-2* and increased the expression of caspas-3, which results in apoptosis. Liu *et al*.[42] have indicated that ATO and 89SrCl2 decrease and increase the expression of *Bcl-2* and *Bax* proteins, respectively. Although the results of the present study are closely confirmed Liu’s findings, 5 µM of ATO+4 Gy did not significantly decrease *Bcl-2* expression compared to 2 µM+4Gy of ATO group (Fig. 4B).

Our findings demonstrated that ATO increased the expression of *Bax* gene at the doses of 2 µM and 5 µM with and without radiation. Nonetheless, combining treatments with 2 µM and 5 µM ATO with 4 Gy of radiation induced significantly more expressions of *Bax* gene (*P*≤0.05; Fig. 4A), suggesting the synergistic effect of ATO and radiation. Several caspases are thought to mediate the early stages of apoptosis. Among them, *caspase-3* is required for the induction of apoptosis by certain effectors[42]. As Figure 5B shows, *caspase-3* is up-regulated by both ATO and radiation in a dose-dependent manner; however, the combination therapy more considerably raised *caspase-3* expression in comparison to the corresponding doses of ATO or IR alone. These results have been confirmed in the other study[43].

Taken together, the findings of this study revealed that ATO in combination with IR more strongly increased the apoptosis progression in U87MG cells, which in turn results in the cell growth arrest and death. Therefore, combined treatment regimen seems to have more inhibiting efficacy compared to single treatment on U87MG cells. Combined treatment decreased the expression level of *Bcl-2* and increased the level of *Bax* and *caspase-3*. These molecular findings show that the designed treatment strategy may induce the intrinsic pathway of apoptosis. Our data not only improved our understanding of the cellular and molecular apoptotic effect of ATO alone or in combination with IR but also proposed a possible therapeutic approach to malignant gliomas, which are famous in resistance to pro-apoptotic therapies. Also, *in vivo* animal studies are required to confirm the potential of ATO for the treatment of GBM cancer.
